# Supplementary material for: Physical activity and risks of breast and colorectal cancer: a Mendelian randomisation analysis
Source: Nat Commun. 2020 Jan 30;11:597. doi: 10.1038/s41467-020-14389-8 (PMC6992637; doi:10.1038/s41467-020-14389-8)
Supplement: Supplementary file 2 — Reporting Summary [file 41467_2020_14389_MOESM2_ESM.pdf]

nature research

Corresponding author(s): Neil Murphy
Last updated by author(s): Dec 19, 2019

Reporting Summary

Nature Research wishes to improve the reproducibility of the work that we publish. This form provides structure for consistency and transparency in reporting. For further information on Nature Research policies, see Authors & Referees and the Editorial Policy Checklist .

Statistics

For all statistical analyses, confirm that the following items are present in the figure legend, table legend, main text, or Methods section.

n/a	Confirmed	
☐	☒	The exact sample size (*n*) for each experimental group/condition, given as a discrete number and unit of measurement
☐	☒	A statement on whether measurements were taken from distinct samples or whether the same sample was measured repeatedly
☐	☒	The statistical test(s) used AND whether they are one- or two-sided<br>*Only common tests should be described solely by name; describe more complex techniques in the Methods section.*
☐	☒	A description of all covariates tested
☐	☒	A description of any assumptions or corrections, such as tests of normality and adjustment for multiple comparisons
☐	☒	A full description of the statistical parameters including central tendency (e.g. means) or other basic estimates (e.g. regression coefficient) AND variation (e.g. standard deviation) or associated estimates of uncertainty (e.g. confidence intervals)
☐	☒	For null hypothesis testing, the test statistic (e.g. *F*, *t*, *r*) with confidence intervals, effect sizes, degrees of freedom and *P* value noted<br>*Give P values as exact values whenever suitable.*
☒	☐	For Bayesian analysis, information on the choice of priors and Markov chain Monte Carlo settings
☒	☐	For hierarchical and complex designs, identification of the appropriate level for tests and full reporting of outcomes
☐	☒	Estimates of effect sizes (e.g. Cohen's *d*, Pearson's *r*), indicating how they were calculated

Our web collection on statistics for biologists contains articles on many of the points above.

Software and code

Policy information about availability of computer code

Data collection	Full data available within paper and supplementary materials
Data analysis	Code available on request

For manuscripts utilizing custom algorithms or software that are central to the research but not yet described in published literature, software must be made available to editors/reviewers. We strongly encourage code deposition in a community repository (e.g. GitHub). See the Nature Research guidelines for submitting code & software for further information.

Data

Policy information about availability of data

All manuscripts must include a data availability statement. This statement should provide the following information, where applicable:

- Accession codes, unique identifiers, or web links for publicly available datasets
- A list of figures that have associated raw data
- A description of any restrictions on data availability

Full data available within paper and supplementary materials

Field-specific reporting

Please select the one below that is the best fit for your research. If you are not sure, read the appropriate sections before making your selection.

☒ Life sciences ☐ Behavioural & social sciences ☐ Ecological, evolutionary & environmental sciences

For a reference copy of the document with all sections, see nature.com/documents/nr-reporting-summary-flat.pdf

Life sciences study design

All studies must disclose on these points even when the disclosure is negative.

Sample size	122,977 breast cancer [69,501 ER positive, 21,468 ER negative] cases and 105,974 controls<br>52,775 colorectal cancer cases and 45,940 controls
Data exclusions	Summary genetic data used for all analyses.
Replication	None
Randomization	Mendelian randomization analyses conducted. These exploit a natural randomization process and this study design is analogous to an RCT
Blinding	N/A

Reporting for specific materials, systems and methods

We require information from authors about some types of materials, experimental systems and methods used in many studies. Here, indicate whether each material, system or method listed is relevant to your study. If you are not sure if a list item applies to your research, read the appropriate section before selecting a response.

Materials & experimental systems

n/a	Involved in the study
☒	☐ Antibodies
☒	☐ Eukaryotic cell lines
☒	☐ Palaeontology
☒	☐ Animals and other organisms
☐	☒ Human research participants
☒	☐ Clinical data

Methods

n/a	Involved in the study
☒	☐ ChIP-seq
☒	☐ Flow cytometry
☒	☐ MRI-based neuroimaging

Human research participants

Policy information about studies involving human research participants

Population characteristics	Summary genetic data from breast and colorectal cancer GWAS consortia. All participants were of European ethnicity.
Recruitment	Multiple studies were included in the GWAS consortia all with variable recruitment strategies. For more information please see the following GWAS publications:<br>Michailidou, K. et al. Association analysis identifies 65 new breast cancer risk loci. Nature 551, 92-94, doi:10.1038/nature24284 (2017).<br><br>Huyghe, J. et al. Discovery of common and rare genetic risk variants for colorectal cancer. Nature Genetics (2018).
Ethics oversight	All individual studies with the GWAS consortia had individual ethical approval.

Note that full information on the approval of the study protocol must also be provided in the manuscript.
